# Editorial of Special Issue “The Interplay of Microbiome and Immune Response in Health and Diseases—2nd Edition”

**DOI:** 10.3390/ijms23137169

**Published:** 2022-06-28

**Authors:** Amedeo Amedei

**Affiliations:** 1 Department of Clinical and Experimental Medicine, University of Florence, 50139 Florence, Italy; amedeo.amedei@unifi.it; 2 SOD of Interdisciplinary Internal Medicine, Azienda Ospedaliera Universitaria Careggi (AOUC), 50139 Florence, Italy

The microbiota refers to the great number of microorganisms (including bacteria, fungi, viruses and parasites) that live on and in humans and has sparked a surge of recent interest. In fact, given that microbial cells in the gut out number host cells, the microbiota influences human physiology, actively impacting on multiple host functions, such as circadian rhythmicity, nutritional processes, metabolism and, finally, immunity [1].

Microbial metabolites, and especially those of the gut microbiota (GM), bridge many, even distant, host zones by way of the immune and hormone systems. For instance, it is currently ascertained that the interplay between the gastrointestinal tract and other organs (e.g., the brain, lung, liver etc., the so-called gut–brain, gut–lung, gut–liver axes) often involves the GM, indicating that the crosstalk between a human and their microbial residents represents a crucial feature for the preservation of a healthy state.

However, the dark side of microbiota is that the its structural and/or functional dysbiosis could severely impact in the development of different pathological conditions, providing new insight for the understanding of diverse diseases, such as IBDs (Inflammatory Bowel Diseases), obesity, autism, stroke, diabetes, and, of course, cancer [2,3].

According to this scenario, the majority of studies on microbiome dynamics look for patterns in which perturbation changes the stable state of animal microbiomes from a healthy one to one is dysbiosis. However, there is also a complementary alternative, as suggested by Zaneveld and colleagues, called as the Anna Karenina principle [4]. They proposed that stochastic microbiological changes brought on by several shocks are what cause community states to shift from stable to unstable. As a result, the microbial community composition of dysbiotic people fluctuates more than that of healthy individuals. In other words, stimuli that impair the host or its microbiome capacity to control community composition cause the Anna Karenina effects, a frequent and significant reaction of animal/human microbiomes to those stressors.

Finally, it is important to keep in mind that the microbiota is not exclusive to the gut. Other organs and host tissues, such as the skin and oral mucosa, are home to both commensal and pathogenic organisms, making it more challenging but undeniably more appealing to investigate the relationship between dysbiosis and human disease.

So, the principal aim of this Special Issue was to evaluate the impact of the different host microbiotas on human health physiology and pathology, welcoming all those studies that will help to well define how microorganisms interact with their hosts, if there are tissue-specific interactions, and what are the effects of their presence, absence, or imbalance.

In this regard, the manuscript of Pistone D. et al. is very interesting. It discusses the recent insights into the “core” and “transient” types of skin microbiota and how their manipulation can contrast skin diseases [5]. For homeostasis and barrier functions to be maintained, the human skin microbiome is crucial. With the collection and cataloguing of more than 10 million bacterial genes, the characterization of its composition and taxonomic diversity has attained impressive goals over time.

The primary dermatological conditions, changes in the microbiota composition linked to them, and suggested methods for skin sample were all covered by the authors. Finally, they focused on topical and dietary probiotics to enhance and maintain skin health, taking into account their potential uses for treating skin conditions.

Another crucial but always neglected microbial community is the oral microbiome, which is the area od focus of Charalambous E.G. et al. [6]. The early-life microbiome (ELM) interacts with the psychosocial environment, in particular during early-life adversity (ELA), defining life-long health trajectories. The ELM also plays a significant role in the right development of the immune system. It is interesting that the authors showed that ELA persists in the oral (buccal and salivary) microbiota for 24 years after exposure to hardship. Additionally, the modifications were linked to an increase in the activation, maturation, and senescence of both innate and adaptive immune cells, while the association was partially reliant on previous herpesvirus exposure and present smoking. In other words, these findings point to various connections between ELA, immunosenescence, and cytotoxicity that develop as a result of ongoing microbiota alterations.

The inflammation/immunity-microbiota axis is also evaluated by the manuscript of Ting-Wei Kao focused on cardiovascular diseases [7]. An increased inflammatory status has recently been proposed as an independent risk factor for developing end-organ comorbidities and adverse prognoses of various chronic illnesses [8]. One of the conditions with intimate linkages to inflammation is cardiovascular disease. In addition to anti-inflammatory drugs, emerging therapeutic approaches include manipulating of the gut microbiota and its metabolites. The mechanism behind how preservation of microbial homeostasis orchestrates cardiovascular physiology needs greater clarification, despite being attractive as a therapeutic target. Consequently, the principal aim of this manuscript was to revisit the role of inflammation from a molecular perspective and assess its implications on clinical outcomes in cardiovascular disease.

As anti-inflammatory drug, Venera Z. Nezametdinova et al. prospected the use of the PFNA operon for inhibiting the “cytokine storm” caused by SARS-CoV-2 as an alternative for anti-TNF-alpha and -IL-6 monoclonal antibodies [9].

One of the main factors in the evolution of the immune systems of many animals was Bifidobacteria. Studies on both animals and humans have recently addressed the issue of specific mechanisms behind the immunomodulatory abilities of Bifidobacteria. Recently, a potential applicant for this position has been identified. All human-gut-dwelling autochthonous Bifidobacterial species have the PFNA cluster, which consists of the five core genes *pkb2*, *fn3*, *aaa-atp*, *duf58*, and *tgm*. The type-III fibronectin-domain-containing protein (FN3) encoded by the I gene and the sensory region of the species-specific serine-threonine protein kinase (PKB2), as well as the transmembrane region of the microbial transglutaminase (TGM), suggest that the PFNA cluster may be involved in the interaction between bacteria and the host immune system. Additionally, the FN3 protein, which is produced by one of the PFNA cluster genes, has cytokine receptor domains and motifs that can specifically bind TNF-alpha. The PFNA cluster might be crucial for detecting immune system signals. The development of anti-inflammatory therapy to treat cytokine storms, one of the worst side effects of SARS-CoV-2 infection, is among the practical implications of this discovery.

So, these studies confirm that the host immune system and microbiota are deeply interrelated. In addition, the inflammatory response derived from the gut microbiota has been linked to several chronic liver diseases, particularly non-alcoholic fatty liver disease (NAFLD), alcohol-related liver disease (ALD), cirrhosis and hepatocellular carcinoma (HCC).

Through a variety of therapies, it is hoped to treat a number of chronic liver illnesses by focusing on damage to the gut–liver axis. In addition to antibiotics, approaches such as fecal microbial transplantation, probiotics, prebiotics, synbiotics, and bacterial metabolites are being investigated. The goal of the manuscript of Sung-Min Won et al. is to explain the GM’s influence on liver illnesses and to pinpoint the connection between the immune response by the gut microbiota and chronic liver diseases [10]. They highlight the function of the gut–liver axis while concentrating on clinical data and treatments for each condition.

In addition to liver disorders, IBDs, as we have previously mentioned, have also been linked with the inflammatory response derived from the gut microbiota. However, little is known about the molecular underpinnings of the cross-talk between the host and the gut microbiota. Serine proteases have been found to be more active in IBD patients, which suggests that they may play a role in the development and maintenance of the illness. The equilibrium between the proteases and the appropriate inhibitors produces the intestinal proteolytic balance. It is interesting to note that whereas the serine protease inhibitors (serpins) encoded by the host have received much reporting, the GM contribution has received little attention. In this scenario, Héla Mkaouar et al. focused on the serpins from the gut microbiota (gut serpinome) and their relevance as a viable therapeutic approach [11], and they offered a succinct overview of the roles of serine protease in IBD physiopathology.

In conclusion, by reporting and discussing these various manuscripts seems very critical the bidirectional communication by host and microbiota that can be mediated through metabolic, immunological, endocrine, and neuronal pathways [12]. However, recently, a new channel of communication through secreted microbiota extracellular vesicles (MEVs) began to appear, as suggested by Salma Sultan et al [13].

MEVs are microscopic, membrane-bound vesicles that carry a variety of biologically active substances across intra- and inter-cellular space. These substances include proteins, mRNA, miRNA, DNA, carbohydrates, and lipids. The biogenesis of microbially produced EVs, their classification and manufacturing processes, as well as their function in inter-bacterial and inter-kingdom signaling, are all thoroughly and discussed in-depth by the authors in this paper.

In other words, the various studies and data provided and reviewed in this Special Issue point to relevance of the microbiota in the formation and maintenance of “health”, as well as in promoting pathological disorders (when the GM function/composition is dysregulated). The complex interactions between humans and microbiota and key biomedical concepts such as “health” and “the individual” raise more than just empirical issues: they also appear to call for the adoption of novel concepts and viewpoints in order to be properly analyzed and applied, particularly for their therapeutic application.

In this context, it is very adequate the contribution of Amedei et al. [14], which illuminates the discussion of the theoretical proposals and innovations (from the ecological component to the notion of the polygenomic organism) aimed at producing this perspective change and analyzing what impact and what new challenges these novel approaches might have on personalized medicine.
